# OLDER MIGRANTS’ EXPERIENCES OF AGING IN PLACE IN HOSTILE AND AUSTERE TIMES

**DOI:** 10.1093/geroni/igae098.1674

**Published:** 2024-12-31

**Authors:** Majella Kilkey, Obert Tawodzera, Jayanthi Lingham

**Affiliations:** University of Sheffield, Sheffield, England, United Kingdom; University of Birmingham, Birmingham, England, United Kingdom; University of Sheffield, Sheffield, England, United Kingdom

## Abstract

Drawing on data from in-depth qualitative interviews with 20 aging-in-place migrants, we explore how they are impacted by the combination of the austerity in welfare state provision and the hostility in migration policy that characterize English society. Informed by the feminist political economy concept of social reproduction, understood as reproducing the population, physically and affectively, in and outside their ‘productive’ working lives (Laslett and Brenner 1989), we find that our research participants are experiencing a ‘crisis of social reproduction’ (Fraser 2016), which strains their social reproductive capacities to breaking point. Our participants are a mix of people who arrived in the post-WW11 era from the British colonies as labor migrants, and people more recently arrived as refugees from conflict-affected parts of Africa. Despite those different migration histories, their current experiences are very similar; a finding we explain through a colonial lens. People who joined England’s ‘productive’ workforce from across the British Empire decades ago are now facing unmet social reproductive needs later in their lives. Meanwhile, others have been driven to the UK due to postcolonial conflict, and as older refugees outside of the ‘productive’ workforce, scant regard is paid to their social reproductive needs. Our findings challenge the dualism prevalent in European research between migration and refugee studies, and suggest the potential of a historically situated colonial lens to understanding older migrants’ experiences. They also highlight the value of analysis of the intersection between migration and welfare policies for deepening understandings of the drivers of ‘crises of social reproduction’.

